# Supplementary material for: Clinical outcomes of immunoglobulin treatment for patients with secondary antibody deficiency: Data from the Ontario immunoglobulin treatment case registry
Source: PLoS One. 2023 Nov 16;18(11):e0294408. doi: 10.1371/journal.pone.0294408 (PMC10653498; doi:10.1371/journal.pone.0294408)
Supplement: Attachment — Submitted filename: Response to reviewers.docx [file pone.0294408.s002.docx]

**Response to reviewers**

Comments by the academic editor:

1. When submitting your revision, we need you to address these additional requirements. Please ensure that your manuscript meets PLOS ONE's style requirements, including those for file naming. The PLOS ONE style templates can be found at <https://journals.plos.org/plosone/s/file?id=wjVg/PLOSOne_formatting_sample_main_body.pdf> and <https://journals.plos.org/plosone/s/file?id=ba62/PLOSOne_formatting_sample_title_authors_affiliations.pdf>.

Response: Thank you. We have made changes accordingly.

2. Thank you for providing the following Funding Statement:
   I have read the journal's policy and the authors of this manuscript have the following competing interests: JC received honoraria and consultation fees from AstraZeneca, Merck, GSK, Sanofi, Genzyme, Takeda, CSL Behring, Octapharma, and Biogen. SB received advisory, speaker and committee fees or research funding from Astria, Canadian Blood Services, CSL Behring, Grifols, Ionis Pharmaceuticals, Kalvista Novartis, Octapharma, Pharvaris, Sanofi, and Takeda. SW received research funding, speaker and consultation fees from CAAIF, ALK Abello, Pfizer, Aimmune Schroeder Foundation, Sean Delaney foundation, GSK, Novartis, CSL Behring, Sanofi, Astrazaneca, Takeda, Teva, Medexus, Mylan, AbbVie, Miravohealth, Bausch Lomb, Avir Pharma and Covis. DWC received consultation and speaker fees from Takeda and CSL Behring. SS and AA have declared that no competing interests exist.
   We note that one or more of the authors is affiliated with the funding organization, indicating the funder may have had some role in the design, data collection, analysis or preparation of your manuscript for publication; in other words, the funder played an indirect role through the participation of the co-authors. If the funding organization did not play a role in the study design, data collection and analysis, decision to publish, or preparation of the manuscript and only provided financial support in the form of authors' salaries and/or research materials, please review your statements relating to the author contributions, and ensure you have specifically and accurately indicated the role(s) that these authors had in your study in the Author Contributions section of the online submission form. Please make any necessary amendments directly within this section of the online submission form. Please also update your Funding Statement to include the following statement: “The funder provided support in the form of salaries for authors [insert relevant initials], but did not have any additional role in the study design, data collection and analysis, decision to publish, or preparation of the manuscript. The specific roles of these authors are articulated in the ‘author contributions’ section.”
   If the funding organization did have an additional role, please state and explain that role within your Funding Statement.
   Please also provide an updated Competing Interests Statement declaring this commercial affiliation along with any other relevant declarations relating to employment, consultancy, patents, products in development, or marketed products, etc.
   Within your Competing Interests Statement, please confirm that this commercial affiliation does not alter your adherence to all PLOS ONE policies on sharing data and materials by including the following statement: "This does not alter our adherence to PLOS ONE policies on sharing data and materials.” (as detailed online in our guide for authors <http://journals.plos.org/plosone/s/competing-interests> ). If this adherence statement is not accurate and there are restrictions on sharing of data and/or materials, please state these. Please note that we cannot proceed with consideration of your article until this information has been declared.

Response: Thank you, we have made the adjustment to our financial disclosure and reported it on the cover letter.

3. We note that you have stated that you will provide repository information for your data at acceptance. Should your manuscript be accepted for publication, we will hold it until you provide the relevant accession numbers or DOIs necessary to access your data. If you wish to make changes to your Data Availability statement, please describe these changes in your cover letter and we will update your Data Availability statement to reflect the information you provide.

Response: Yes, all data will be provided upon acceptance.

4. Please review your reference list to ensure that it is complete and correct. If you have cited papers that have been retracted, please include the rationale for doing so in the manuscript text, or remove these references and replace them with relevant current references. Any changes to the reference list should be mentioned in the rebuttal letter that accompanies your revised manuscript. If you need to cite a retracted article, indicate the article’s retracted status in the References list and also include a citation and full reference for the retraction notice.

Response: Thank you, this has been revised. No articles used in reference in our article were retracted.

Reviewer’s comments:

Reviewer #1: I write regarding PONE-D-23-25320 entitled "Clinical Outcomes of Immunoglobulin Treatment for Patients with Secondary Antibody Deficiency: Data from the Ontario Immunoglobulin Treatment Case Registry ". An observational study evaluating immunoglobulin replacement therapy in patients with secondary antibody deficiency. A total of 141 patients were evaluated and especially their quality of life and infection rates were evaluated. Indeed, the use of IgRT in clinical practice is controversial in terms of cost effectiveness and indications. Therefore, I think that this article meets the unmet need on an important issue. I have a few suggestions.

1. Firstly why were patients recruited in only 2 years (June 2020 -September 2022)?

Response: Patient recruitment for the case registry is still ongoing as part of our program (Ontario Immunoglobulin Treatment program). However, to analyze data for this manuscript, Sep 30, 2022, was indicated as a cut-off date for data extraction.

2. In total, there were 141 patients in the study. While there were 9 patients using IVIG and 131 using SCIG, the other 1 patient?

Response: Our sincere apology of this error. The one patient was initially included because of secondary antibody deficiency criterion; however, we realized that the patient was never started on immunoglobulin replacement therapy by the time of data extraction. Therefore, we decided to remove the patient. The total number of patients is now 140, with 131 on SCIG and 9 on IVIG.

3. It would be more appropriate to specify the references after the point rather than before.

Response: There were a few references written within the sentence, these were corrected.

4. Which questionnaire was used as quality of life? should be specified by reference. Was the questionnaire used validated in your country?

Response: The data included in this manuscript does not contain quality of life data. We changed the terminology of “quality of life” to “general health” where we asked if the patient felt that his/her overall health was the same, worse, or better at the time of study visit as compared to before IgRT.

5. The long version of SCIG should be written on line 83.

Response: Thank you. It has been corrected.

6. In Table-1, n should also be specified in the IgRT section.

Response: Thank you. We have specified n in Table-1 as suggested.

7. Who should make the statistics of the article? If not, you should seek help from a professional statistician. It was observed that parametric values were averaged.

Response: We consulted a statistician. Median and interquartile range is reported for data that did not have a normal distribution.

Reviewer #2: Secondary immune disorders are far more prevalent and can be caused by various diseases and their treatment, certain medications and sometimes due to surgical procedures. Secondary antibody deficiencies are generally poorly defined and there are no guidelines for managing patients with this condition. In this respect, the study evaluating a large number of patients is important. In addition, the importance of national and international databases, where demographic, clinical data, treatment options and outcomes of patients with rare diseases are recorded, should be emphasized again.

There are some issues that require minor revision.

1. The number of patients is 141, 131 on SCIg, 9 on IVIg. Which route for Ig replacement is given to the remaining 1 patient?

Response: Our sincere apology of this error. The one patient was initially included because of secondary antibody deficiency criterion; however, we realized that the patient was never started on immunoglobulin replacement therapy by the time of data extraction. Therefore, we decided to remove the patient. The total number of patients is now 140, with 131 on SCIG and 9 on IVIG.

2. The mean age of the study group is 66 years. What is the age range?

Response: Age range is between 23- 85 years old.

3. Vaccination responses to specific vaccines (the pneumococcal polysaccharide vaccine, anti-Hib,..) should be checked to give an indication regarding immune function, even if the patient, especially with hematologic malignancy, has not suffered from infections, as they may be at risk of severe sepsis. Did the authors check the vaccine responses before the initiation of IgG therapy?

Response: We followed the guidelines by starting secondary antibody deficient patients on IgRT when IgG are low, especially <4g/L or when there is a history of recurrent sinopulmonary infections (European Medicines Agency. Guideline on core SmPC for human normal immunoglobulin. Committee for Medicinal Products for Human Use). The results of the pneumococcal and hemophilus serology tests are not widely available in Ontario, unfortunately. The current turnaround time for the pneumococcal serology test result is more than one year in our region. This is not useful in clinical setting.

4. What were the indications for IgRT in patients with normal IgG levels?

Response: Normal IgG levels at our sites are between 7.0 and 16.0 g/L. Only two patients had normal IgG levels prior to initiation of therapy. The indication for treatment of the first patient was low IgG2 and low IgG3 levels. The patient was on chronic immunosuppressive therapy for severe scleroderma affecting both lungs and having undergone double lung transplant within a few years after starting IgRT. The other patient had an abnormal protein electrophoresis showing abnormal band in IgG kappa, with total gamma globulins of 5.0 despite total IgG showing a level of 8.6 g/L before IgRT.

5. Did any of the patients experience severe bacterial infections (sepsis, osteomyelitis, meningitides,..) before and after Ig treatment?

Response: This is a very good point. There was a limitation in data collection for 131 patients who were already on IgRT prior to ONIT case registry enrollment. The data collected did not specify type of infections. We only recorded the overall number of infections, treatments for infections, ED visits, and hospitalizations based on patient’s report which was subjected to a recall bias. However, once the patients were in our program, we prospectively recorded types and severity of infections. We did not have any reported sepsis, osteomyelitis, or meningitides after starting on IgRT.

6. Did any of the patients have accompanying severe or chronic complications (bronchiectasis, malnutrition, renal impairment…)??

Response: Thank you. Two patients had concurrent bronchiectasis; ten patients had renal impairment. This was added to the results section under demographics.

7. Were there any patients with protein loss (renal or intestinal)?

Response: This is a good point. None of the 140 patients reported in this manuscript had evidence of renal or intestinal protein loss. In our patient population, the cause of secondary antibody deficiency is attributed to their primary hematologic malignancy or immune dysregulation and the immunosuppressive therapy associated with it.

8. Did the authors make a statistical analysis to correlate the infection rates and neutropenia prior to IgRT?

Response: This is also a good point. Of 140 patients, only 83 had neutrophil counts prior to IgRT recorded. We graphed a scatter plot and analyzed the correlation between reported infection rate and neutrophil counts before IgRT. We found no correlation with spearman r = -0.075 [95% CI -0.292 – 0.1494]. This data should be confirmed prospectively in IgRT naïve patients who are newly enrolled in our ONIT registry going forward.

9. The authors should emphasize the formulations of the chosen SCIg (10%, 16%, 20%, or facilitated 10%).

Response: This was added to the results section under IgRT dosage, formulations, and treatment duration.
